# Progress of the Cholera in Europe and Asia

**Published:** 1847-12

**Authors:** 


*Progress of the Cholera in Europe and Asia.*——Foreign papers announce that this dreadful scourge is again making its appearance in different parts of Europe and Asia. Its approach is creating alarm with those who have watched its progress from the plains of Scinde towards Western Europe. About eighteen months since, it ravaged the banks of the Indus with awful severity, inflicting serious loss upon the British troops at Kurrachee and Hyderabad. About the same time it raged in Affghanistan; spread from thence into Persia, which it traversed from east to west, spreading to the northward into Tartary, and southwardly into Turkish Kurdistan, and the pachalic of Bagdad. Early in the present year, it made its appearance to the west of the Caucasian mountains, and committed great ravages in the Russian army acting against the Circassions; and we just now learn of its re-appearance in Europe, having broken out at Taganrog, Marianopolis, and other ports on the westerly shores of the sea of Azof, Smolensk, Riga, Tifflis, Kars, Kontais and Trebizond. Great alarm is felt at Warsaw, where the authorities were preparing hospitals. As in its former progress towards Europe, in the years 1830 and 1831, the general course of the pestilence has been nearly due north-west; and it seems, so far, to have travelled at about the same rate as on that occasion. In 1831 it made its appearance on the shores of the Baltic (at Riga. Dantzic and Memel), in the month of may, at Vienna and Berlin in August, at Hamburgh in October, and reached England in the beginning of November.—*Boston Med. Sur. Jour.*

